# The Effect of Different Extraction Methods on Extraction Yield, Physicochemical Properties, and Volatile Compounds from Field Muskmelon Seed Oil

**DOI:** 10.3390/foods11050721

**Published:** 2022-02-28

**Authors:** Huijun Zhang, Yushu Yuan, Xiuxiu Zhu, Runzhe Xu, Huishan Shen, Qian Zhang, Xiangzhen Ge

**Affiliations:** 1 School of Life Science, Huaibei Normal University, Huaibei 235000, China; zhhuijun@126.com (H.Z.); yuanyushu123@163.com (Y.Y.); z17856579357@163.com (X.Z.); xrz1577594325@163.com (R.X.); 2 College of Food Science and Engineering, Northwest A&F University, Xianyang 712100, China; 18202949392@163.com (H.S.); z-grace@nwafu.edu.cn (Q.Z.)

**Keywords:** field muskmelon seed oil, extraction method, physicochemical properties, volatile compound, fatty acid, antioxidant

## Abstract

Field muskmelon seed oil was extracted by press extraction (PE), Soxhlet extraction (SE), organic extraction (OSE), and aqueous extraction (AE). The oils were then evaluated for their physicochemical properties, fatty acid composition, volatile compounds, and antioxidant properties. A high yield oil was found in the SE sample. The AE sample had the highest elevated acid and peroxide values, while PE and OSE had the highest oil iodine content. The oil samples did not differ significantly in their fatty acid profile depending on the extraction method. However, E-nose, HS-GC-IMS, and HS-SPME-GC-MS showed that the flavor composition of the four samples was significantly different, attributed to the changes in the composition and content of the compounds caused by the different extraction methods. Furthermore, the strongest FRAP and the free radical scavenging ability of DPPH and ABTS^+^ showed in the SE sample. In general, SE’s seed oil has certain advantages when applied to the muskmelon seed oil industry.

## 1. Introduction

China is the world’s largest importer of edible vegetable oil, with a self-sufficiency rate of less than 40%, so there is an urgent need to increase the domestic oil production capacity [1]. Melon seed oil can be used as a raw material to prepare biodiesel and edible oil. Field muskmelon (*Cucumis melo* L. var. agrestis Naud.) is a plant of the Cucurbitaceae genus Cucumis, native to Africa and widely distributed in India, Sudan, China, and other places. It is eaten as a vegetable when it is immature and as a fruit when ripe. Field muskmelon seeds contain a lot of fat, which can be squeezed into oil for consumption. Ji et al. [2] extracted the oil of field muskmelon seeds through the pressing method and the oil yield reached 25%. The main component of field muskmelon seed oil is triacylglycerols and it can become a good source of γ-tocopherol and linoleic acid [3].

Generally, different extraction methods may result in differences in physicochemical properties obtained from oil. The hot-pressing process needs to go through high temperature procedures, which could promote the increase of specific aroma components to a certain extent. At the same time, it will also destroy some heat-sensitive active ingredients. To ensure complete extraction, Soxhlet extraction is designed according to repeated solvent reflux and solvent siphoning. However, this method has disadvantages, such as a long extraction time and large amount of organic waste.

In addition, solvent extraction is a commonly used traditional lipid extraction method. In this process, organic solvents can damage plant cell walls or disrupt the interaction between the lipids and the tissue matrix to extract oil. Since the oil extracted in the solvent extraction is likely to have organic solvents, it is an excellent strategy to replace the organic solvents with water, as in the extraction solvent aqueous method. However, the aqueous extraction method can easily form emulsified oil, resulting in a low extraction rate. In addition, cold pressing [4], supercritical fluid extraction [5], and ultrasonic/microwave-assisted extraction [6] are also widely used in the extraction of seed oil. Alrashidi et al. [7] found that the Soxhlet method had a higher oil yield than cold pressing in Al-Qassim nigella oil. Zhang et al. [8] found that different extraction methods significantly affected milk thistle seed oils’ minor component content and antioxidant activity.

Volatile components are an essential factor affecting the sensory properties, quality, and consumption of oils. Electronic nose (E-nose) and gas chromatography-mass spectrometry (GC-MS) technology are the most commonly used to analyze volatile substances. With the development of modern instrumental analysis technology, gas chromatography-ion mobility spectrometry (GC-IMS) has been applied to analyze volatile substances as a new analytical technique. This technology identifies the ions based on the migration rate of different gas-phase ions in the gas phase in the electric field. The result shows the flavor difference of different samples with intuitive and visual fingerprints. Combining multiple technologies can play a good role in verification and complementation [9]. The combination of various methods has been applied to determine different varieties of oil [10], processing methods [11], and adulteration, etc. [12]. This study extracted field muskmelon seed oil using four extraction methods. Their physicochemical properties, volatile flavor components, and antioxidant activity were comprehensively analyzed to provide specific scientific data for the research and industrial application of the extraction method of field muskmelon seed oil.

## 2. Materials and Methods

### 2.1. Materials

Field muskmelon (Cucumis bisexualis) was collected in Lixin County (116°12′48″ E 33°9′5.50″ N), Anhui Province. The fruit diameter of the field muskmelon was between 5.87 and 4.74 cm, and the average fruit weight was 40–45 g. The seeds were obtained by removing the peel and pulp, and then washed clean and baked at 50 °C for 24 h (moisture content was 7.8 ± 0.14%). The length and width of the seeds were 4–5 mm and 2.0–2.4 mm, respectively, with pointed apex, rounded base. The field muskmelon seeds were crushed by a universal crusher (TLG-10-08-04, Beijing Tianli Hengcheng Technology Co., Ltd., Beijing, China). The seeds were sifted through a sieve and a granulation of 800–400 μm was used for the experiments.

### 2.2. Extraction Method of Oil

Pressing extraction (PE): The 100 g sample was hot pressed with a DH-50 screw oil press (the temperature of the pressing chamber was set to 90 °C). It was composed of infinite threads in a cylinder, and the oil was extracted from the seeds by friction between the threads and the wall of the cylinder.

Organic solvent extraction (OSE): The 100 g sample was added in 1000 mL n-hexane. The sample was placed in a magnetic stirrer (85-2B, Qiuzuo Technology Co., Ltd., Shanghai, China) and extracted at 55 °C for 3 h. The suspension was then filtered with filter paper. A rotary vacuum evaporator recovered the n-hexane at 50 °C. To further reduce the residual solvent, the extracted oil was placed in a vacuum drying oven at 50 °C until constant weight.

Soxhlet extraction (SE): The 10 g sample was put into a paper sleeve and placed in a Soxhlet extractor with a capacity of 250 mL. Petroleum ether was used as the solvent, and the reaction was carried out at 40 °C for 6 h. After the extraction, the sample was rotated to remove the solvent and placed in a vacuum drying oven to a constant weight.

Aqueous extraction (AE): The 100 g seed powder was added with distilled water at a material-to-liquid ratio of 1:10, placed in a magnetic stirrer (85-2B, Qiuzuo Technology Co., Ltd., Shanghai, China) and extracted at 50 °C for 3 h. Then the sample was centrifuged (5000× *g*) for 20 min and the supernatant oil and emulsified layer were transferred to a 50 mL centrifuge tube. The samples were then placed in a refrigerator at −20 °C for 24 h, the sample was taken out and thawed at room temperature for demulsification, and the upper oil layer was taken.

### 2.3. Extraction Yield

After the extractions, the total yield of the extracted oil was determined according to the following equation:

(1) $$\mathrm{Extraction}\;\mathrm{yield}\left(\%\right)=m_{1}/m_{0}\times 100$$

where *m*_1_ is the total mass of the extracted oil, and *m*_0_ is the initial mass of the seeds used in each extraction.

### 2.4. Quality Indexes

The acid value, iodine value, peroxide value, and saponification value of the samples were determined according to AOCS Official method from the American Oil Chemist’s Society [13].

(2) $$\mathrm{Peroxide}\;\mathrm{value}\;\left({\mathrm{meq}\;O}_{2}/\mathrm{kg}\;\mathrm{oil}\right)=\frac{\left(V_{1}-V\right)\times N}{M}$$

where *V*_1_ = the volume of Na_2_S_2_O_3_ used for the titration of the sample (mL); *V* = the amount of the Na_2_S_2_O_3_ used for the titration of blank (mL); *N* = the normality of Na_2_S_2_O_3_ (mol/L); and *M* = the weight of the sample (g).

The acid value was calculated according to the formula as follows:

(3) $$\mathrm{Acid}\;\mathrm{value}\;\left(\mathrm{mgKOH}/g\right)=\frac{\left(V-V_{0}\right)\times c\times 56.1}{m}$$

where *V* = volume of standard potassium hydroxide consumed (mL); *V*_0_ = the amount of the potassium hydroxide used for the titration of blank (mL); *N* = molarity of standard potassium hydroxide solution (mol/L); and *m* = the weight of the sample (g).

### 2.5. Fatty Acid Composition

Methyl esterification: The 1 g sample was added to 8 mL 0.5 mol/L potassium hydroxide-methanol solution and then refluxed on a water bath at 80 °C for 1 h. The 7 mL of 15% boron trifluoride methanol solution was added and refluxed for 2 min. After quickly cooling to 25 °C, the mixture was mixed with 30 mL of n-heptane, shaken for 2 min, and then a saturated 2 mL sodium chloride aqueous solution was added. The 5 mL of the n-heptane extraction solution was sucked out, and 5 g of anhydrous sodium sulfate was added. The upper solution was sucked into the sample bottle for determination.

Chromatographic conditions: HP-5MS 60 m × 0.25 mm × 0.25 µm) capillary column; heating program: initial column temperature 100 °C, kept for 1 min; increased at 5 °C/min to 200 °C, held for 2 min; then increased at 3 °C/min and kept 280 °C for 3 min; the split ratio was 1:50. Mass spectrometry conditions: ionization mode was electron impact ion source (EI source); electron energy was 70 eV; transmission line temperature was 280 °C; ion source temperature was 230 °C; quadrupole temperature was 150 °C; solvent delay 2.6 min; full scan mode (SCAN), scan range 50~1000 u. The fatty acid composition was identified by comparing retention times to known standards. The quantity of fatty acids was calculated by the peak area normalization law and represented as the relative percentage of each fatty acid to the area of total fatty acids.

### 2.6. Comprehensive Analysis of Volatile Aroma Compounds of Seed Oil Samples

#### 2.6.1. E-Nose

The 0.5 g oil sample was added into a 30 mL E-nose headspace bottle and room temperature equilibration was performed for 20 min. E-nose (PEN3, Airsense Analytics GmbH, Schwerin, Germany) parameters: sample preparation: 5 s, detection time: 60 s, automatic zero adjustments: 5 s, cleaning time 300 s, gas flow rate: 400 mL/min.

#### 2.6.2. Headspace Solid Phase Microextraction Gas-Mass Spectrometry (HS-SPME-GC–MS)

An amount of 2 ± 0.01 g per sample was placed in a 20 mL vial. Divinylbenzene/carboxene/polydimethylsiloxane (DVB/CAR/PDMS, 50/30 µm coating, 1 cm length) fiber (Supelco Inc., Bellefonte, PA, USA) was used for headspace sampling. The sample vials were equilibrated in the incubator at 60 °C for 30 min under agitation at 500 rpm. After 30 min of extraction at 50 °C, the fiber was immediately desorbed into the GC-MS injection port at 250 °C for 3 min.

HS-SPME-GC–MS analysis of the seed oil samples was performed on Shimadzu GCMS-QP-2010 plus system. DB-17MS capillary chromatographic column (60 m × 0.25 mm × 0.25 µm) was used for the analysis. The operating conditions of the column were as follows: 40 °C was kept for 3 min, then the temperature was increased to 240 °C at 4 °C/min and held for 10 min. The inlet temperature was 250 °C. The carrier gas was He, and the flow rate was 1 mL/min. The mass scan range of m/z was 40–600, and the ion source temperature was 230 °C. Analysis was performed by comparing the spectrum with the MS NIST14 library (NIST14, version 2.2, National Institute of Standards and Technology, Gaithersburg, MD, USA). The peak area normalization method was used for a relative quantitative calculation to obtain the relative percentage content of each compound in oil samples of different extraction methods.

#### 2.6.3. Headspace Solid Phase Microextraction Ion Mobility Spectroscopy (HS-SPME-GC-IMS

In brief, 2 g of the oil sample was weighed and placed into a 20 mL headspace glass sampling vial. Subsequently, samples were incubated at 60 °C for 30 min. After incubation, 500 μL of headspace was automatically injected into the injector (45 °C, splitless mode) using a heated syringe at 65 °C.

A GC-IMS system (Flavourspec^®^, G.A.S, Dortmund, Germany) equipped with an Rtx-WAX capillary column (15 m × 0.53 mm × 1.0 μm, RT-12,424) was employed for this study. Headspace sampler detection conditions: headspace furnace incubation temperature: 70 °C, incubation time: 15 min, sample injection volume: 500 μL, vibration speed: 500 r/min, injection needle temperature: 85 °C.

IMS conditions: column type: FS-SE-54 CB-1 (15 m × 0.53 mm, 0.53 μm); column temperature: 60 °C; carrier gas: high purity N2; positive ion mode; drift tube length: 9.8 cm; tube linear voltage: 500 V/cm; drift tube temperature: 45 °C. The GC-IMS equipment came with instrument analysis software (Laboratory Analytical Viewer (LAV) and plug-ins (Reporter, Gallery Plot) and GC-IMS Library Search qualitative analysis software) to collect and analyze the volatile components of seed oil. The peak area normalization method was used for a relative quantitative calculation to obtain the relative percentage content of each compound in the oil samples of different extraction methods.

### 2.7. Tocopherols

Tocopherols content was carried out with HPLC according to the method described by Dimić et al. [4]. The sample in n-hexane solution (1%, *m*/*v*) was injected into the chromatographic system. The analysis was performed using a 1200 series liquid chromatograph manufactured by Agilent Technologies (Palo Alto, CA, USA), equipped with a fluorescence detector. The separation was done on a LiChrospher Si 60 (250 mm × 4 mm, 5 μm) column (Merck, Darmstadt, Germany). The fluorescence detector was set at 290 nm excitation wavelength and 330 nm emission wavelength. Peaks were identified separately based on retention times determined for α-, β-, and γ-tocopherol standards (Merck, Darmstadt, Germany). The amounts of tocopherols in the extracts were calculated as mg tocopherols in a 100 g oil sample.

### 2.8. Antioxidant Activity

The oil sample was mixed with absolute ethanol to obtain 10, 20, 30, 40, and 50 mg/mL sample diluent to determine antioxidant activity. All results were determined using a UV spectrophotometer (N6000, Shanghai Youke Instrument Co., Ltd., Shanghai, China).

#### 2.8.1. DPPH Radical Scavenging Ability

An amount of 3 mL of the sample solution was mixed with 3 mL of DPPH solution (2,2 diphenyl-1-picrylhdrazyl, 0.1 mM). After reacting for 30 min, the absorbance was measured at a wavelength of 517 nm. Anhydrous ethanol replaced the test solution to determine the absorbance as control [14]. Obtained results were reported as mmol of Trolox equivalents per 100 g of oil sample.

#### 2.8.2. ABTS^+^ Radical Scavenging Activity

ABTS^+^ radical solution (5 mL of 7.4 mM 2,2′-azinobis (3-ethylbenzothiazoline-6-sulfonic acid) solution and 5 mL of 2.6 mM potassium persulfate solution) was incubated 14 h without light and at room temperature. The working solution was mixed with absolute methanol and its light absorption value at 734 nm was required to reach 0.7 ± 0.02. The 3 mL of the sample solution and 6 mL of ABTS^+^ working solution were mixed and reacted for 30 min. The mixture was measured by absorbance at a wavelength of 734 nm. Obtained results were reported as mmol of Trolox equivalents per 100 g of oil sample.

#### 2.8.3. Ferric Reducing Antioxidant Power (FRAP) Assay

FRAP was determined according to Ma et al. [15] with some modifications. The sample (50 μL) was added to 2 mL fresh FRAP working solution (mixture of 300 mmol/L acetate buffer solution, 10 mmol/L 2,4,6-tri(2pyridyl)-s-triazine (TPTZ) solution and 20 mmol/L FeCl_3_ solution in a ratio of 10:1:1). The mixture was incubated at 37 °C for 5 min in dark conditions. The absorbance at 593 nm was measured. The final result was expressed as the concentration of antioxidants having a ferric reducing ability equivalent to that of 1 mM L^−1^ FeSO_4_, based on the standard curve for FeSO_4_ × 7H_2_O at a concentration range between 100 and 1000 uM L^−1^.

### 2.9. Data Analysis

All experimental results were expressed as mean value and standard deviation of repeated trials (three times for all) and determined using Microsoft Excel 2016 software (Microsoft Corporation, Redmond, WA, USA). Duncan’s multiple tests were used for statistical analysis, and the difference was considered significant at 95% (*p* < 0.05).

## 3. Results and Discussion

### 3.1. Yield and Oil Quality Indexes

The effect of the extraction methods on the yield and physicochemical properties of field muskmelon seed oil is shown in Table 1. SE had the highest oil yield (34.47%), which indicated that Soxhlet extraction was the most effective technique for extracting vegetable oil [16]. The next highest extraction rates were 24.64% and 22.90%, by OSE and PE, respectively. The yield of the AE method was significantly lower than the other three methods (18.57%). The extraction rate of the different extraction methods was quite different, which was consistent with the research results of Péres et al. [17]. Both SE and OSE use the principle of interaction between organic solvents and tissue lipids to extract oil. The reasons for the higher extraction rate in the SE sample might be (1) the sample phase was repeatedly contacted with a fresh solvent to ensure complete extraction; (2) the heat applied to the distillation flask extended to the extraction chamber to a certain extent to keep the system in a higher temperature [18]. PE is an extraction process that relies on external forces to mechanically break the walls of plant cells and force oil to be squeezed out of plant cells. The high residual oil rate of the oil cake caused by insufficient extrusion in this process was the main reason for the low extraction rate. A large amount of emulsion was produced when using AE, which affected the separation of free oil, resulting in a lower extraction rate [19].

There were relatively large differences in the oil quality indexes of the field muskmelon seed oil extracted by different methods (Table 1). When the acid value is high, the oil will have some adverse effects, such as lowering the oil’s smoke point, lowering the oxidation stability, and being prone to rancidity during storage, which will bring abnormal taste stimulation [20]. In addition, peroxide value can reflect the degree of oxidation of fats and fat-containing substances, fatty acids, etc. [21,22]. Finally, the iodine value reflects the degree of unsaturation of vegetable oils.

The AE samples had the highest acid and peroxide values. This was because the extraction process was performed in a pH-adjusted water system and prolonged exposure to air promoted the oxidation of the samples [23]. The higher peroxide value in the PE sample may be due to the frictional heat generated by the screw, which accelerated the oxidation process of the grease [24]. The saponification value of the OSE sample was the highest. In contrast, that of the PE sample was relatively low, indicating that the molecular weight of fatty acids in the OSE sample was greater than in the other methods. The high iodine content of oil obtained from SE means that the degree of unsaturation was maintained.

Tocopherols are natural antioxidant compounds that stabilize oils. The contents of tocopherols in the samples extracted by different methods are shown in Table 1. The γ-tocopherol is the main tocopherol in field muskmelon seed oil, ranging from 7.56 mg/100 g oil to 20.63 mg/100 g oil. The γ-tocopherol inhibits lipid oxidation in foods by stabilizing hydroperoxide and other free radicals. The highest content of γ-tocopherol was in the SE, while the lowest was in the AE. The order of the content of α- and β-tocopherol obtained by the four extraction methods was SE > AE > OSE > PE, indicating that the extraction method was an important factor affecting the composition of tocopherols. The effects of different extraction methods on the content of tocopherols were also found in grape seeds and green coffee oil [4,20].

### 3.2. Fatty Acid Composition

Table 2 shows the fatty acid composition of four samples in the field muskmelon seed oil extracted using different methods. Nine fatty acids (five saturated fatty acids, two monounsaturated fatty acids, and two polyunsaturated fatty acids) were detected in the oil samples. However, the fatty acid content of different samples varied greatly. This was due to the different responses of the properties or structures of fatty acids to different extraction conditions, such as pressure, solvent type, temperature, etc. [25].

In general, the fatty acid profile was characterized by a high proportion of unsaturated fatty acids (UFAs), especially a high proportion of monounsaturated fatty acids (MUFAs), which was similar to previously reported studies [2]. Oleic acid (C18:1) was the primary fatty acid in all seed oil samples, followed by linoleic acid (C18:2). Appropriately increasing the intake of oleic acid and linoleic acid in the daily diet could effectively reduce the occurrence of hypercholesterolemia and cardiovascular disease [26,27], which revealed that field muskmelon seed oil had a high nutritional value. The oleic acid content (C18:1) in the OSE samples was the highest (62.12%). This can be explained by the polarity of the solvent, which has a higher affinity for oleic acid [28]. The linoleic acid (C18:2) content in the PE samples was the highest at 26.60%, and the OSE content was the lowest at 17.36%. In addition, the PE samples contained high levels of palmitic acid (C16:0). The higher content of saturated fatty acid from the PE and OSE methods was due to the oxidation of unsaturated fatty acids that are easily oxidized due to high temperature and long-term air exposure [20].

### 3.3. E-nose Measures the Odor Profile

**Figure 1:**
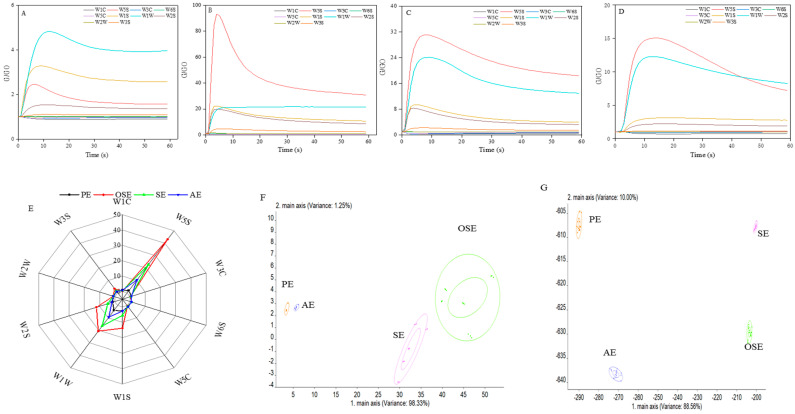
Effects of different extraction methods on E-nose responses of field muskmelon seeds oil. E-nose responses for representative press extraction (**A**); organic extraction (**B**); Soxhlet extraction (**C**); aqueous extraction (**D**); sensor response radar diagram (**E**); PCA sample distribution diagram (**F**); LDA sample distribution diagram (**G**).

The E-nose is based on a series of chemical gas sensors combined with multivariate statistical methods, using 10 sensors to profile and identify different odor components [29]. Therefore, Figure 1A–D could intuitively compare the response value of the E-nose to the seed oil extracted by the different methods. Among them, the W5S (nitrogen oxide compounds), W1W (sulfides), W1S (methyl compounds), and W2S (alcohols, aldehydes and ketones) sensors had the strongest response value in all seed oil samples, indicating that four components are the main volatile flavor components making up the seed oil.

PCA reduces the dimensionality of the E-nose data, while the LDA is a statistical method that uses the signals of all sensors to study the category of samples [30]. Figure 1F is a PCA two-dimensional image of four seed oil E-nose data. The contribution rates of the first and second principal components were 98.33% and 1.25%, respectively, and the total contribution rate was 99.58%, which covers all the sample information. There was no overlap between the four groups of samples, indicating some differences in volatile components. This was similar to different extraction methods in golden melon seed oil [31]. In LDA, the contribution rates of linear discriminant functions LD1 and LD2 were 88.56% and 10.00%, respectively, and the total contribution rate was 98.56% (Figure 1G). There was no overlap between the four groups, which achieves the purpose of distinguishing samples. These differences may be due to the formation of new volatile compounds and other similar volatile compounds under different extraction methods. Studies have shown that different extraction methods can identify and distinguish seed oil using E-nose technology.

### 3.4. HS-SPME-GC-MS

Volatile components are an essential feature that determines people’s acceptance and preference for the quality of edible vegetable oils, and they play a vital role in the overall flavor of seed oils. Flavor substances in seed oils mainly include small molecular weight alcohols, aldehydes, ketones, esters, pyrazines, furans, and pyrroles produced by the lipoxygenase pathway branched-chain amino acid degradation, Maillard reaction, thermal decomposition of sugars (proteins), and the degradation of vitamins [32].

A total of 63 volatile substances were identified, including 7 chemical classes of volatile substances: hydrocarbons (21), esters (6), aldehydes (6), alkenes (4), ketones (8), alcohols (6), acids (3), and others (9) (Table 3). This was similar to the results of seed oil volatile flavor components reported in golden melon seeds [31], camellia seeds [33], and sesame seeds [34], which reflects that the scent of the seed oil is not reflected by one or several compounds, but by the synergistic effect of multiple components to reflect different characteristic scents.

Alkane compounds are mainly derived from the homolysis of fatty acid alkoxy radicals, which give seed oil an inherent refreshing fragrance [9]. AE, SE, and OSE samples all had a higher content of alkane volatile substances, 89.43, 91.81, and 55.79%, respectively. Especially in SE, where the most kinds of alkanes were found (16), among which, the content of n-pentane (58.45%) was the highest, indicating that the SE sample scent was mainly based on refreshing mint fragrance. However, the content of hydrocarbons was significantly reduced in the PE sample, which may be due to the conversion of part of the hydrocarbons into intermediates forming heterocyclic compounds during the preheating process. The flavor threshold of olefins is generally low, with aromas such as spicy, woody, citrus, camphor, lemon, and tropical fruit aromas. The content of olefins in the OSE samples was the highest, which was 33.43%. Among them, styrene was the olefinic compound with the highest content in the OSE sample, an aromatic monomer that gave the sample a slightly sweet taste. The olefins content in the AE samples was the lowest (1.42%).

Ester compounds generally have wine, floral, and typical fruit aromas and are essential. Ester compounds were detected in four samples (1.33% in the AE sample, 0.14% in the SE sample, 5.59% in the PE sample, and 6.09% in the OSE sample). (S)-l-Alanine ethylamide was an ester substance shared by AE and SE, and docosanoic acid nonyl ester, decanoic acid, decyl ester, and dibutyl phthalate were unique to PE.

Aldehydes are mainly derived from the oxidative decomposition of unsaturated fatty acids. Most aldehyde volatile substances have a low odor threshold, so those volatile substances have a relatively significant contribution to the overall aroma of seed oil. Among the four different extraction methods of field muskmelon seed oil samples, the PE samples had the most types and content of aldehyde volatile substances, accounting for 35.12% of the total. The higher contents of aldehydes were hexanal (oil fragrance, green grass fragrance), pentanal (almond, malt, pungent), and decanal (sweet fragrance, citrus fragrance, wax fragrance, floral fragrance), which made outstanding contributions to the aroma components of the PE samples. The AE samples contained three kinds of aldehydes, which accounted for 3.90% of the total volatile components. No aldehydes were found in the OSE samples. In the OSE process, the transition of single bonds at both ends of the substances is easier to break into esters, hydrocarbons and alkenes (Table 3), and depends on the extraction rate process.

Generally, the Maillard reaction generates ketones or further aldehydes oxidation [35]. Most ketones have a unique fragrance, creamy or fruity scent, and the aroma is excellent and lasting. The PE samples had the highest ketone content at 22.45%, followed by the OSE samples (2.19%) and the AE samples (1.54%), and the SE samples were the lowest (0.39%). The highest ketone content in the PE samples was 2-Methyl-3-heptanone, at 14.51%.

Alcohols have a soft odor, showing a delicate, light, sweet, mellow, wine, and fruity aroma. Linear saturated alcohols have a higher threshold and have little effect on flavor, while monounsaturated alcohols have a lower threshold and a more significant contribution to taste [36]. In the four samples, six alcohols were detected in the PE samples, accounting for 16.80% of the total volatile compounds. Only one (Eucalyptol) was detected in AE and OSE, and no alcohols were found in the SE samples. In the PE samples, the highest content of the alcohols was n-octanol, which was a component with a strong aromatic odor. This is due to the heat treatment during the pressing process with high temperature conditions resulting in the thermal degradation of sugar or the Maillard reaction. In addition, the Soxhlet extraction process was not conducive to the retention of alcohols.

Most acids are precursors or reaction products of other flavors. In the four samples, only acids were detected in PE, namely butyric acid (0.82%), hexanoic acid (2.59%), and myristic acid (0.53%). Hexanoic acid is mainly produced by the enzymatic hydrolysis reaction in the lipoxygenase pathway. It has a cheese and oily smell and was high in the PE samples, which may have a particular influence on the green scent of field muskmelon seed oil.

**Figure 2:**
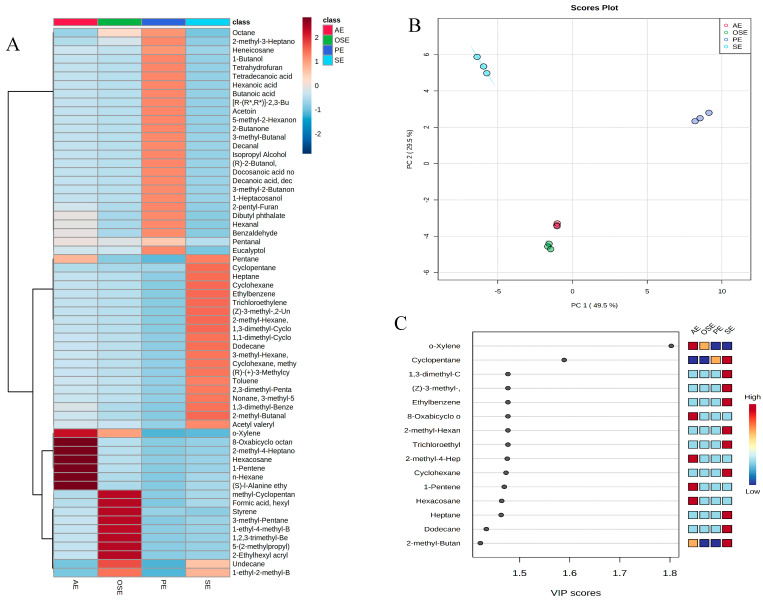
Multivariate statistical analysis volatiles by HS-PME-GC-MS identified on field muskmelon seed oil samples. (**A**): heatmap cluster based on the normalized quantities of the identified volatiles; (**B**): PLS-DA score plot of chemical components in field muskmelon seed oil; (**C**): VIP scores in PLS-DA.

In the PLS-DA score plot of chemical composition, the four extraction method seed oil samples were discriminated according to the two principal components with the cumulative contribution rate of 79.00% (Figure 2B). Representing a weighted sum of squares of the PLS weight, the VIP with value > 1 is usually considered an important and potential chemical marker to the model being studied. A total of 15 compound variables were accounted for with significant contribution (VIP > 1), and O-xylene showed the greatest VIP in all samples (Figure 2C).

### 3.5. HS-GS-IMS

#### 3.5.1. Identification of Volatile Compounds in Seed Oil

**Figure 3:**
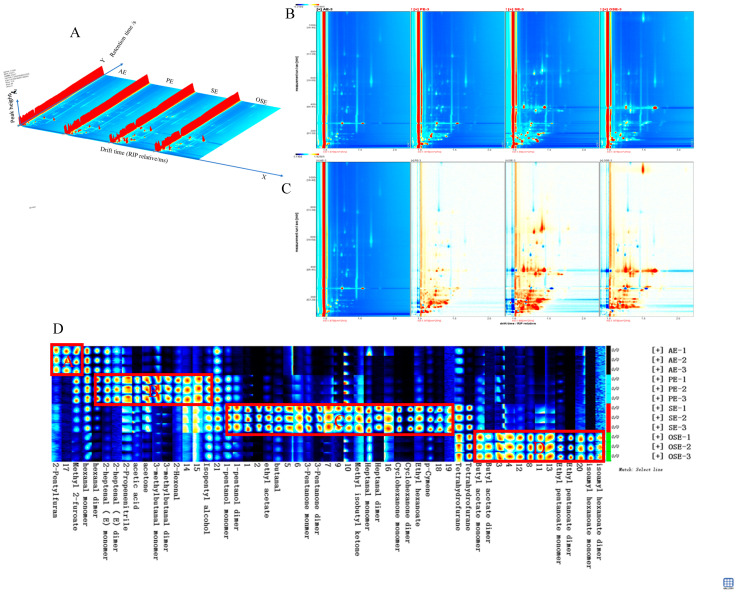

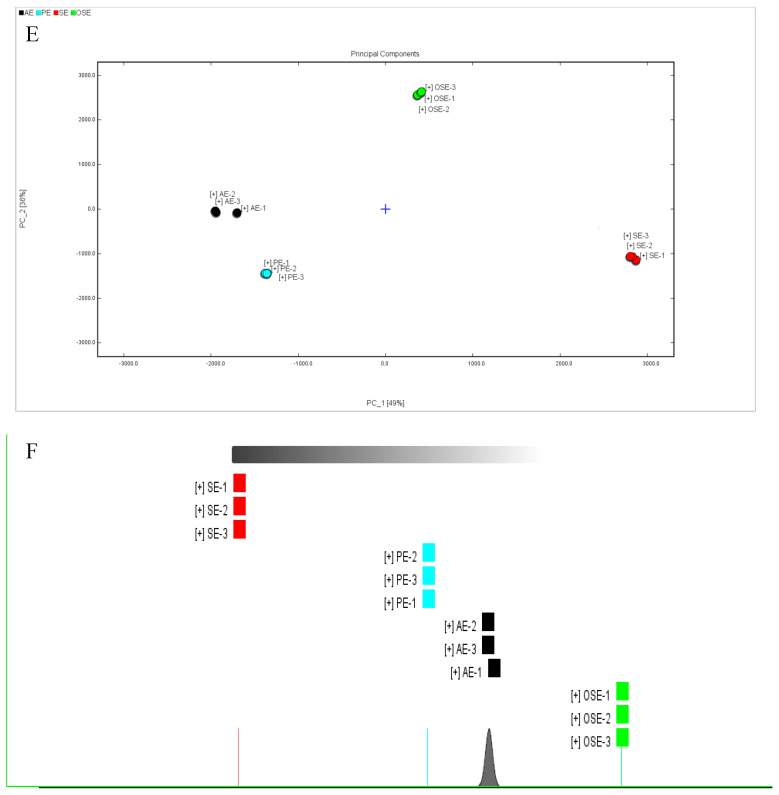
Volatile compounds analysis by HS-GC-IMS. (**A**) 3D-topographic; (**B**) topographic plots; (**C**) topographic subtraction plots; (**D**) volatile compounds fingerprint comparisons. Each row represents all the signals selected in a sample. Each column represents the signals of the same volatile compounds. “M” and “D” denote monomer and dimer, respectively. (**E**) principal component analysis; (**F**) fingerprint similarity analysis.

HS-GS-IMS was used to analyze the volatile compounds of the seed oil samples with different extraction methods. Figure 3A is the 3D spectra of the volatile components of different samples. Each point on both sides of the ion peak represents an explosive compound, and the shade of the color indicates the level of content. From external observations, the GC-IMS three-dimensional spectra of the four essential oil samples were very similar, and it was difficult to distinguish the volatile components visually.

Projecting the 3D GC-IMS spectrum of Figure 3A onto a two-dimensional plan top view is shown in Figure 3B, which can directly compare and analyze the differences of the volatile components of the four seed oil samples. As shown in Figure 3B, the red vertical line at the abscissa 1.0 is the reaction ion peak (RIP). Each point on either side of the RIP peak represents a compound, and the color reflects the concentration of the substance (blue indicates a low concentration, and red indicates a high concentration). It could be observed that most of the signals appeared in the retention time of 100–800 s and the drift time of 1.0–2.0 (relative to RIP), and the volatile components of the sample were well-separated (Figure 3B). Based on the spectra of the seed samples prepared from the AE samples, there were prominent red and blue spots in the spectra of the three kinds of seed oils by different extraction methods (Figure 3C), that is, the composition of volatile substances was different for each of the extraction methods.

#### 3.5.2. Fingerprint Profile Comparisons in Seed Oil Samples by Different Extraction Methods

To comprehensively and intuitively analyze the differences in the composition of volatile substances in the four samples, the volatile substance signal peaks in the spectrum of each sample were selected to form the above fingerprint. A compound may produce 1–2 or more spots (representing monomer, dimer, or trimer), depending on the concentration and nature of the volatile components [11].

HS-GC-IMS detected 55 peaks (containing monomers and dimers), including 33 typical compounds, but there were still 21 compounds with no qualitative results due to limited data in the library database. According to the identified compounds, the volatile compounds in seed oils were alcohols (3), aldehydes (10), ketones (6), esters (9), furans (3), etc., which are slightly different from SPME-HS-GC-MS analysis results.

To further analyze the differences in the main volatile compounds, the signal peaks of the four seed oil samples were compared with fingerprints (Figure 3D). Hexanal (monomer) had a higher response in these four samples, indicating that no matter what method was used to extract the seed oil, it presented birth fat, grass smell, and apple aroma. Among them, the A region substance could be used as the characteristic volatile substance of the AE sample, and its content was much higher than that in other samples. From left to right, there were mainly 2-pentylfuran and 2-methyl furoate. Substances in the B area could be used as the characteristic volatile substances of the PE samples, including hexanal (multimer), E-2-heptanal (monomer), E-2-heptanal (multimer), 2-acrylonitrile, acetic acid, acetone, 3-methylbutyraldehyde (monomer), 3-methylbutyraldehyde (multimer), 2-hexanal, and isoamyl alcohol, etc. C area substances could be used as the characteristic volatile substances of SE seed oil, including n-pentanol (monomer), n-pentanol (multimer), ethyl acetate, butyraldehyde, 3-pentanone (Monomer), 3-pentanone (multimer), methyl isobutyl ketone, heptaldehyde (monomer), heptaldehyde (multimer), cyclohexanone (monomer), cyclohexanone ketones (polymers), ethyl caproate, and 4-isopropyl toluene, etc. The characteristic volatile substances of the OSE samples were shown in area D, mainly including butyl acetate (monomer), butyl acetate (polymer), ethyl valerate (monomer), valeric acid ethyl (polymer), isoamyl caproate (monomer), and isoamyl caproate (multimer), etc. The C area had the most extensive range, indicating that the characteristic response of the flavor compounds in the SE samples was the highest.

To highlight the difference among extraction methods for volatile compounds in seed oil, PCA and FSA (Figure 3E) were performed based on the area signal intensity of the identified compounds. The cumulative variance contribution rates of PC1 and PC2 accounted for 48% and 35%, respectively, and all seed oil samples showed significant differences. FSA (Figure 3F) can be used to analyze the similarity of compound fingerprints by calculating and comparing Euclidean distance. The larger the sample distance in the figure, the more pronounced the sample difference. The FSA results further confirmed the analysis conclusions from the PCA results.

### 3.6. Antioxidant Properties

**Figure 4:**
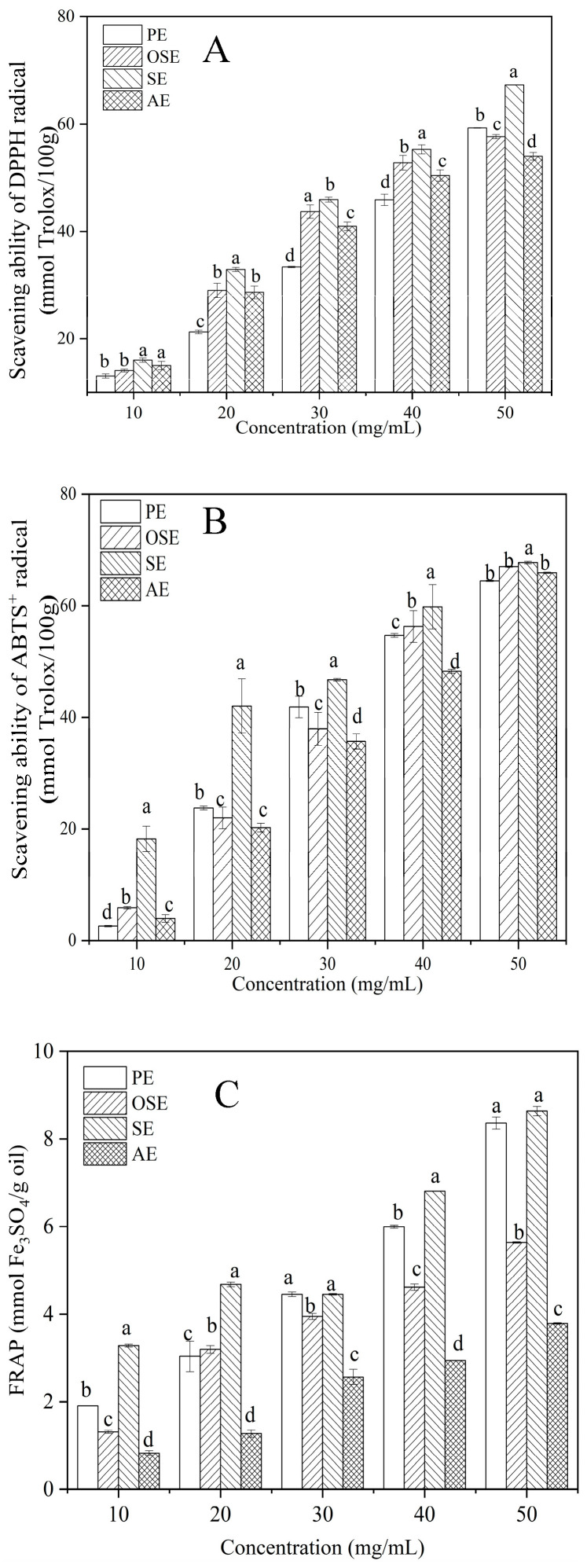
The antioxidant activities of field muskmelon oil samples. (**A**): DPPH radical scavenging ability; (**B**): ABTS^+^ radical scavenging activity; (**C**): ferric reducing antioxidant power assay. PE: press extraction; SE: Soxhlet extraction; OSE: organic extraction; AE: aqueous extraction. Bars represent the mean values of antioxidant activities. Vertical bars represent standard error (±SE). Different letters represent significant differences (*p* < 0.05).

The DPPH radical scavenging activities steadily increased with the seed oil content increase. As a result, the SE sample had the strongest DPPH radical scavenging activity (16.00–67.32 mmol Trolox/100 g), followed by AE and OSE, and PE (13.03–59.29 mmol Trolox/100 g) was the weakest (Figure 4A). Similarly, the highest ABTS radical scavenging activity in seed oil content was observed in SE (18.22–64.61 mmol Trolox/100 g), followed by AE and PE, and the lowest was the OSE sample (16.97–43.83 mmol Trolox/100 g) (Figure 4 B). Meanwhile, for the FRAP (Figure 4 C), the PE and SE samples exhibited a narrower range (1.90–8.36 mmol Fe_3_SO_4_/g oil, 3.28–8.63 Fe_3_SO_4_/g oil) compared with the AE (0.83–3.79 Fe_3_SO_4_/g oil) and the OSE (1.31–5.64 Fe_3_SO_4_/g oil) samples.

Field muskmelon seed oil is rich in natural antioxidants. These active ingredients mainly include fatty acids, polyphenols, flavonoids, tocopherols, sterols, carotene, and β-carotene, etc. [2]. Extraction methods have varying degrees of influence on the antioxidant capacity of seed oil. Chouaibi et al. [37] found that the red pepper seed oils extracted via the Soxhlet sample had the lowest DPPH and ABTS^+^ scavenging values compared with cold pressing and supercritical-CO2’s extracted sample. On the other hand, Ma et al. [15] demonstrated that pepper seed oil obtained by pressure extraction had the highest antioxidant capacity using FRAP. In contrast, the conventional solvent extraction sample had the greatest increase in DPPH radical-scavenging activity. The extracted oils exhibited better or similar antioxidant activity in comparison to seed oils from nutmeg (16.46–31.69 μM/g), white mustard (4.21–7.39 μM/g), anise (3.44–12.52 μM/g), coriander (2.24–4.96 μM/g), and caraway (6.32–8.81 μM/g) depending on the solvent [38]. Previous papers indicated that there might be two kinds of antioxidant capacity of oil: (1) the difference in oil composition, mainly in the composition and ratio of unsaturated fatty acids, and (2) the difference in the content of natural antioxidant substances in the oil, such as tocopherols and other substances [15]. The complex composition and synergistic effect of antioxidant compounds in the sample horse pear seed oil may be why it has the highest antioxidant capacity in determining DPPH and ABTS. In our samples, due to the high temperature of PE and the acid-base effect in the AE process, the antioxidant activities of these seed oil samples were lower than the other extraction methods.

## 4. Conclusions

This study obtained field muskmelon seed oil from different extraction methods (press extraction, Soxhlet extraction, organic extraction, and aqueous extraction). The oils obtained using Soxhlet extraction had significant advantages in extraction rate and antioxidant capacities, and aqueous extraction samples owned the highest acid and peroxide values. In contrast, aqueous extraction samples had higher oil iodine content. The proportion of unsaturated fatty acids in all oil samples was very high, with oleic acid and linoleic acid being the main ones. W5S, W1W, W1S, and W2S in E-nose analysis were the primary sensors to distinguish the flavor characteristics of the seed oil samples. In addition, 63 volatile compounds were detected through HS-SPME-GC-MS analysis, and HS-GC-IMS identified 55 volatile compounds. E-nose, HS-GC-MS, and GC-IMS combined with some multivariate data analysis showed that these four samples had different volatile compounds. In conclusion, Soxhlet extraction is a suitable way for field muskmelon seed oil to be obtained and has better performance in the oil yield and quality.

## Figures and Tables

**Table 1:** Yield and oil quality indexes of field muskmelon seed oils ^A,B^.

Determination	**Oils**			
	PE	OSE	SE	AE
Extraction yield (%)	22.90 ± 0.24 ^bc^	24.64 ± 0.14 ^b^	34.47 ± 0.39 ^a^	18.57 ± 0.39 ^c^
Acid value (mg KOH/g)	0.676 ± 0.010 ^b^	0.224 ± 0.001 ^d^	0.448 ± 0.157 ^c^	2.349 ± 0.004 ^a^
Peroxide value (m eq O_2_/kg)	1.18 ± 0.05 ^b^	0.58 ± 0.10 ^c^	0.67 ± 0.09 ^c^	3.61 ± 0.35 ^a^
Saponification value (mg KOH/g)	191.91 ± 5.90 ^b^	223.52 ± 6.76 ^a^	215.44 ± 3.54 ^a^	227.42 ± 6.32 ^a^
Iodine value (g I/100 g)	104.42 ± 0.04 ^c^	114.71 ± 0.96 ^b^	126.48 ± 1.13 ^a^	111.82 ± 0.37 ^b^
α-Tocopherol (mg/100 g)	1.25 ± 0.03 ^d^	1.33 ± 0.21 ^c^	2.14 ± 0.12 ^a^	1.88 ± 0.06 ^b^
β-Tocopherol (mg/100 g)	1.25 ± 0.05 ^d^	1.55 ± 0.01 ^c^	2.71 ± 0.17 ^a^	2.21 ± 0.06 ^b^
γ-Tocopherol (mg/100 g)	11.26 ± 1.30 ^c^	14.79 ± 1.18 ^b^	20.63 ± 0.14 ^a^	7.56 ± 0.08 ^d^

^A^ PE: press extraction; SE: Soxhlet extraction; OSE: organic extraction; AE: aqueous extraction. ^B^ Values are the means of three replicates ± standard deviation. Means with different letters within a row are significantly different at *p* < 0.05.

**Table 2:** Fatty acid composition of field muskmelon seed oils. ^A,B^.

Property	Content (%)			
	PE	OSE	SE	AE
Saturated fatty acid (SFAs)				
Palmitic acid (C16:0)	7.00 ± 0.54 ^a^	2.73 ± 0.25 ^c^	5.01 ± 0.01 ^b^	2.62 ± 0.17 ^c^
Stearic acid (C18:0)	6.78 ± 0.22 ^a^	3.62 ± 0.14 ^b^	3.64 ± 0.47 ^b^	2.82 ± 0.11 ^c^
Behenic acid (C22:0)	6.14 ± 0.47 ^b^	2.83 ± 0.14 ^d^	10.16 ± 0.77 ^a^	3.51 ± 0.15 ^c^
Lignoceric acid (C24:0)	1.37 ± 0.02 ^c^	1.61 ± 0.24 ^b^	0.65 ± 0.04 ^d^	1.87 ± 0.07 ^a^
Eicosanoic acid (C20:0)	0.84 ± 0.00 ^c^	5.76 ± 0.44 ^a^	0.22 ± 0.0 ^d^	4.78 ± 0.41 ^b^
Unsaturated fatty acid (UFAs)				
Monosaturated Fatty Acid (MUFAs)				
Oleic acid (C18:1)	49.60 ± 0.45 ^b^	62.12 ± 0.14 ^a^	55.61 ± 0.52 ^b^	54.99 ± 1.14 ^b^
Palmitoleic acid (C16:1)	1.02 ± 0.23 ^c^	1.47 ± 0.04 ^b^	0.11 ± 0.00 ^d^	2.73 ± 0.11 ^a^
Polysaturated fatty acids (PUFAs)				
Linoleic acid (C18:2)	26.60 ± 0.32 ^a^	17.36 ± 0.78 ^c^	24.07 ± 0.88 ^a^	23.26 ± 1.03 ^b^
Linolenic acid (C18:3)	0.67 ± 0.08 ^c^	2.51 ± 0.10 ^b^	0.53 ± 0.03 ^c^	3.43 ± 0.17 ^a^
SFAs	22.12 ± 2.36 ^a^	16.55 ± 1.14 ^c^	19.68 ± 0.25 ^b^	15.59 ± 0.23 ^d^
MUFAs	50.62 ± 0.52 ^c^	63.59 ± 1.23 ^a^	55.72 ± 0.47 ^b^	57.71 ± 0.25 ^b^
PUFAs	27.27 ± 1.53 ^a^	19.86 ± 0.23 ^b^	24.60 ± 0.22 ^c^	26.70 ± 1.47 ^a^
PUFA/SFA	1.23 ± 0.04 ^b^	1.20 ± 0.03 ^b^	1.25 ± 0.47 ^b^	1.71 ± 0.23 ^a^

^A^ PE: press extraction; SE: Soxhlet extraction; OSE: organic extraction; AE: aqueous extraction. ^B^ Values are the means of three replicates ± standard deviation. Means with different letters within a row are significantly different at *p* < 0.05.

**Table 3:** The flavor composition of field muskmelon seed oils ^A,B^.

Compound Name	CAS	RI	Molecular Formula	Content (%)			
				PE	OSE	SE	AE
Hydrocarbons							
Pentane	109-66-0	518	C_5_H_12_		1.03 ± 0.08	58.45 ± 1.56	26.00 ± 0.45
3-Methyl-pentane	96-14-0	554	C_6_H_14_		13.11 ± 0.28	0.36 ± 0.08	0.37 ± 0.01
N-Hexane	110-54-3	618	C_6_H_14_			5.21 ± 0.12	62.34 ± 3.21
Nethyl-cyclopentane	96-37-7	661	C_6_H_12_		39.64 ± 0.25	3.92 ± 0.01	0.27 ± 0.01
8-Oxabicyclo octane	286-45-3	850	C_7_H_12_O	1.44 ± 0.12			0.15 ± 0.01
Hexacosane	630-01-3	2606	C_26_H_54_				0.30 ± 0.03
Cyclopentane	287-92-3	600	C_5_H_10_	1.62 ± 0.05		11.16 ± 0.04	
Cyclohexane	110-82-7	618	C_6_H_14_			2.66 ± 0.24	
2-Nethyl-hexane,	591-76-4	653	C_7_H_16_			1.78 ± 0.04	
2,3-Dimethyl-pentane,	565-59-3	589	C_7_H_16_			0.74 ± 0.07	
1,1-Dimethyl-cyclopentane	1638-26-2	734	C_7_H_14_			0.14 ± 0.00	
3-Dethyl-hexane,	589-34-4	653	C_7_H_16_			1.89 ± 0.45	
1,3-Dimethyl-cyclopentane	2532-58-3	722	C_7_H_14_			0.86 ± 0.14	
Heptane	142-82-5	717	C_7_H_16_			1.38 ± 0.01	
Cyclohexane, methyl-	108-87-2	781	C_7_H_14_			0.54 ± 0.07	
Undecane	1120-21-4	1115	C_11_H_24_		1.15 ± 0.04	0.98 ± 0.11	
Dodecane	112-40-3	1214	C_12_H_26_			1.22 ± 0.08	
Nonane, 3-methyl-5-propyl-	629-59-4	1413	C_13_H_28_			0.52 ± 0.01	
Octane	111-65-9	816	C_8_H_18_	1.48 ± 0.47	0.52 ± 0.01		
Heneicosane	629-94-7	2109	C_21_H_44_	0.87 ± 0.11			
5-(2-Methylpropyl)-Nonane	62185-53-9	1185	C_13_H_28_		0.34 ± 0.01		
Sum				5.41 ± 0.45	55.79 ± 1.07	91.81 ± 0.97	89.43 ± 1.45
Esters							
(S)-l-Alanine ethylamide	17344-99-9	864	C_5_H_11_NO_2_			0.09 ± 0.00	0.87 ± 0.01
Dibutyl phthalate	84-74-2	2037	C_16_H_22_O_4_	2.41 ± 0.12			0.46 ± 0.0.2
Formic acid, hexyl ester	629-33-4	981	C_7_H_14_O_2_		0.41 ± 0.00	0.05 ± 0.00	
Docosanoic acid nonyl ester	42233-05-6	3270	C_31_H_62_O_2_	0.50 ± 0.01			
Decanoic acid, decyl ester	1654-86-0	2177	C_20_H_40_O_2_	2.68 ± 0.04			
2-Ethylhexyl acrylate	103-11-7	1208	C_11_H_20_O_2_		5.68 ± 0.35		
Sum				5.59 ± 0.25	6.09 ± 0.35	0.14 ± 0.01	1.33 ± 0.21
Aldehydes							
2-Methyl-butanal	96-17-3	643	C_5_H_10_O			1.31 ± 0.11	0.08 ± 0.01
Hexanal	66-25-1	806	C_6_H_12_O	23.07 ± 0.14			3.48 ± 0.24
Benzaldehyde	100-52-7	982	C_7_H_6_O	2.02 ± 0.01			0.34 ± 0.01
3-Methyl-butanal	590-86-3	643	C_5_H_10_O	4.87 ± 0.48			
Pentanal	110-62-3	707	C_5_H_10_O	2.99 ± 0.24			
Decanal	112-31-2	1204	C_10_H_20_O	2.17 ± 0.25			
Sum				35.12 ± 0.57	0.00	1.31 ± 0.11	3.90 ± 0.33
Alkenes							
1-Pentene	109-67-1	508	C_5_H_10_		0.10 ± 0.01		0.42 ± 0.14
Styrene	100-42-5	883	C_8_H_8_	2.34 ± 0.23	33.33 ± 0.54		1.00 ± 0.07
(Z)-3-Methyl-2-undecene	19780-34-8	1199	C_12_H_24_			0.65 ± 0.01	
Trichloroethylene	1979/1/6	734	C_2_HCl_3_			2.12 ± 0.24	
Sun				2.34 ± 23	33.43 ± 0.78	2.77 ± 0.36	1.42 ± 0.18
Ketones							
2-Methyl-4-heptanone	626-33-5	888	C_8_H_16_O				1.54 ± 0.05
Acetyl valeryl	96--04-8	989	C_7_H_12_O_2_			0.06 ± 0.00	
(R)-(+)-3-Methylcyclopentanone	6672-30-6	832	C_6_H_10_O			0.33 ± 0.04	
5-Methyl-2-hexanone	110-12-3	789	C_7_H_14_O	4.43 ± 0.11			
3-Methyl-2-butanone	563-80-4	590	C_5_H_10_O	1.88 ± 0.01			
2-Butanone	78-93-3	555	C_4_H_8_O	0.89 ± 0.04			
Acetoin	513-86-0	717	C_4_H_8_O_2_	0.74 ± 0.02			
2-Methyl-3-heptanone	13019-20-0	888	C_8_H_16_O	14.51 ± 0.07	2.19 ± 0.88		
Sum				22.45 ± 0.19	2.19 ± 0.88	0.39 ± 0.04	1.54 ± 0.05
Alcohols							
Isopropyl alcohol	67-63-0	482	C_3_H_8_O	3.50 ± 0.44			
(R)-2-Butanol,	14898-79-4	581	C_4_H_10_O	2.68 ± 0.04			
1-Butanol	71-36-3	662	C_4_H_10_O	5.46 ± 0.48			
[R-(R*,R*)]-2,3-Butanediol	24347-58-8	743	C_4_H_10_O_2_	0.57 ± 0.04			
1-Heptacosanol	2004-39-9	2948	C_27_H_56_O	2.91 ± 0.57			
Eucalyptol	470-82-6	1059	C_10_H_18_O	1.68 ± 0.22	0.29 ± 0.01		0.28 ± 0.00
Sum				16.8 ± 1.01	0.29 ± 0.01	0.00	0.28 ± 0.00
Acids							
Butanoic acid	107-92-6	775	C_4_H_8_O_2_	0.82 ± 0.06			
Hexanoic acid	142-62-1	974	C_6_H_12_O_2_	2.59 ± 0.45			
Tetradecanoic acid	544-63-8	1769	C_14_H_28_O_2_	0.53 ± 0.06			
Sum				3.94 ± 0.57	0	0	0
Other							
2-Pentyl-furan	3777-69-3	1040	C_9_H_14_O	7.24 + 0.48			0.61 ± 0.01
Tetrahydrofuran	109-99-9	589	C_4_H_8_O	1.11 ± 0.04			
1,3-Dimethyl-benzene	108-38-3	907	C_8_H_10_			1.85 ± 0.22	0.19 ± 0.01
O-Xylene	95-47-6	907	C_8_H_10_		0.97 ± 0.12		1.30 ± 0.14
Toluene	108-88-3	794	C_7_H_8_			0.99 ± 0.04	
Ethylbenzene	100-41-4	893	C_8_H_10_			0.19 ± 0.04	
1-Ethyl-2-methyl-benzene	611-14-3	1006	C_9_H_12_		0.73 ± 0.11	0.55 + 0.09	
1-Ethyl-4-methyl-benzene	622-96-8	1006	C_9_H_12_		0.19 ± 0.01		
1,2,3-Trimethyl-benzene	526-73-8	1020	C_9_H_12_		0.32 ± 0.1		
Sum				8.35 ± 0.55	2.21 ± 0.21	3.58 ± 0.01	2.10 ± 0.22

^A^ PE: press extraction; SE: Soxhlet extraction; OSE: organic extraction; AE: aqueous extraction. ^B^ Values are the means of three replicates ± standard deviation.

## Data Availability

The data presented in this study are available on request from the corresponding author.
